# Diffuse Large B-Cell Lymphoma Presenting as Clinically Isolated Massive Splenic Disease: A Case Report

**DOI:** 10.7759/cureus.117673

**Published:** 2026-10-09

**Authors:** Abhishek Mohanty, Ali Z Anwar, Sunil Kumar, Amit Minz

**Affiliations:** 1 General Surgery, Shahid Nirmal Mahato Medical College, Dhanbad, IND; 2 Surgical Oncology, Shahid Nirmal Mahato Medical College, Dhanbad, IND

**Keywords:** diffuse large b-cell lymphoma, extranodal non-hodgkin lymphoma, massive splenomegaly, r-chop chemotherapy, splenectomy

## Abstract

Diffuse large B-cell lymphoma (DLBCL) presenting as clinically isolated massive splenic disease is uncommon and may pose substantial diagnostic and therapeutic challenges. We report a 47-year-old woman who presented with six months of progressive left upper quadrant pain, abdominal fullness, anorexia, early satiety, intermittent dyspnea, fatigue, fever, night sweats, and a 7 kg unintentional weight loss. Examination revealed massive splenomegaly without peripheral lymphadenopathy. Laboratory investigations showed leukocytosis with neutrophilic predominance and elevated lactate dehydrogenase. Contrast-enhanced computed tomography (CT) demonstrated a markedly enlarged spleen measuring approximately 37 × 25 × 15 cm without significant thoracic, abdominal, or pelvic lymphadenopathy. Because of severe mass effect symptoms, extreme splenic enlargement, and the need for definitive tissue diagnosis, elective open splenectomy was performed. The spleen measured 40 × 27 × 12 cm and weighed 7.5 kg. Histopathology demonstrated diffuse effacement of splenic architecture by large atypical lymphoid cells. Because immunohistochemical facilities were unavailable at the treating institution, representative paraffin-embedded tissue blocks were evaluated at an external laboratory by a hematopathologist. Immunohistochemistry demonstrated CD20, CD79a, and PAX5 positivity; CD3 and CD5 negativity; CD10 and BCL6 positivity with MUM1 negativity; a Ki-67 proliferation index of approximately 80%; and MYC and BCL2 expression below commonly used double-expressor thresholds. These findings confirmed DLBCL of germinal center B-cell phenotype involving the spleen. Postoperative contrast-enhanced CT of the chest, abdomen, and pelvis showed no significant nodal or additional visceral disease, supporting a clinically localized splenic presentation. Pretreatment and post-treatment 18F-FDG PET-CT and bone marrow assessment were not performed because of resource and logistical limitations; therefore, occult systemic or marrow involvement could not be definitively excluded, and PET-based metabolic response could not be assessed. The patient received six cycles of rituximab, cyclophosphamide, doxorubicin, vincristine, and prednisone (R-CHOP) every 21 days, beginning 28 days after surgery. At 18 months after completion of chemotherapy, she remained clinically well with no evidence of recurrent disease on surveillance clinical examination and CT. This case emphasizes the importance of adequate tissue diagnosis, external expert immunophenotypic confirmation when required, careful systemic staging, and individualized consideration of open splenectomy in patients with symptomatic extreme splenomegaly.

## Introduction

Primary splenic lymphoma (PSL) is an uncommon extranodal lymphoma characterized predominantly by involvement of the spleen, with reported incidence varying according to the diagnostic criteria used [1,2]. Among splenic lymphomas, diffuse large B-cell lymphoma (DLBCL) represents an important aggressive histological subtype and may present with rapidly progressive splenomegaly, constitutional symptoms, abdominal discomfort, and systemic inflammatory manifestations [2,3]. Although splenic involvement may occur as part of disseminated lymphoma, lymphoma presenting predominantly within the spleen without clinically evident lymphadenopathy or other visceral disease is distinctly uncommon. Establishing whether splenic disease represents a primary splenic presentation or secondary involvement by an otherwise occult systemic lymphoma can therefore be challenging, particularly when complete staging investigations are not feasible.

The clinical presentation of splenic lymphoma is frequently nonspecific. Patients may present with left upper quadrant pain, abdominal distension, early satiety, weight loss, fever, night sweats, and fatigue. In advanced cases, progressive splenic enlargement can produce substantial mass effect symptoms and may occupy a large proportion of the abdominal cavity. Massive splenomegaly also creates a broad differential diagnosis that includes hematological malignancies, myeloproliferative neoplasms, infections, infiltrative disorders, storage diseases, and other lymphoproliferative conditions [3,4]. Cross-sectional imaging, particularly contrast-enhanced computed tomography (CT), is valuable for defining splenic size and morphology and for evaluating regional lymph nodes and additional visceral involvement; however, imaging findings alone are generally insufficient to establish the histological subtype of lymphoma.

Definitive diagnosis requires adequate tissue for histopathological and immunophenotypic evaluation. Image-guided splenic biopsy has emerged as a useful diagnostic option in appropriately selected patients and can avoid diagnostic splenectomy in many circumstances [5]. Nevertheless, the feasibility and risk-benefit profile of percutaneous biopsy depend on the patient's anatomy, splenic size, lesion characteristics, vascularity, and the amount of tissue required for comprehensive pathological classification. In patients with extreme splenic enlargement and significant mass effect symptoms, splenectomy may occasionally be considered when less invasive tissue acquisition is unsuitable, inconclusive, or unlikely to provide adequate tissue, while simultaneously providing symptomatic relief. Importantly, splenectomy should not be regarded as a substitute for systemic lymphoma-directed therapy in DLBCL.

Histopathological classification of DLBCL requires integration of morphology with immunophenotypic findings. Immunohistochemistry is essential for establishing B-cell lineage and for further subclassification, including assessment of germinal center versus activated B-cell phenotype and expression of markers relevant to prognostic stratification [2,6,7]. In settings where specialized hematopathology and immunohistochemistry are not available locally, external expert pathological assessment may be required to ensure accurate diagnosis and classification.

## Case presentation

A 47-year-old woman presented to the Department of General Surgery with a six-month history of progressively worsening left upper quadrant abdominal pain, abdominal fullness, progressive abdominal distension, anorexia, early satiety, intermittent dyspnea, and generalized fatigue. The pain was dull, nonradiating, predominantly localized to the left hypochondrium, aggravated after meals and prolonged standing, and partially relieved by rest. During the preceding three months, she had experienced an unintentional weight loss of approximately 7 kg, associated with intermittent fever with chills and night sweats. She also reported constipation. There was no history of nausea, vomiting, jaundice, hematemesis, melena, urinary symptoms, or significant alteration in bowel habits. She had no previous history of diabetes mellitus, hypertension, tuberculosis, chronic liver disease, autoimmune disease, or malignancy. There was no history of previous abdominal surgery or long-term medication use. She denied smoking, alcohol consumption, and recreational drug use. There was no significant family history of hematological or other malignancies.

On examination, the patient was conscious, oriented, and hemodynamically stable. Mild pallor was present. There was no icterus, cyanosis, clubbing, pedal edema, or palpable peripheral lymphadenopathy. Abdominal examination revealed marked fullness of the left upper abdomen, with a massively enlarged spleen occupying almost the entire left hemiabdomen and extending across the midline toward the right iliac fossa. The spleen was firm, smooth-surfaced, mildly tender, and moved with respiration. No hepatomegaly, ascites, or other palpable intra-abdominal mass was identified. Cardiovascular, respiratory, and neurological examinations were unremarkable.

Initial laboratory evaluation revealed a hemoglobin level of 12.2 g/dL and a total leukocyte count of 21,000/mm³, with neutrophilic predominance. The differential leukocyte count showed neutrophils 78% (16,380/mm³), lymphocytes 14% (2,940/mm³), monocytes 5% (1,050/mm³), eosinophils 2% (420/mm³), and basophils 1% (210/mm³). Serum lactate dehydrogenase (LDH) was elevated at 480 U/L (reference range, 140-280 U/L). The erythrocyte sedimentation rate was 45 mm/hour, and C-reactive protein was 24 mg/L. Liver and renal function tests, serum electrolytes, and coagulation parameters were normal. Screening serological tests for HIV-1/2, hepatitis B surface antigen, anti-hepatitis C virus antibodies, Epstein-Barr virus immunoglobulin M, and cytomegalovirus immunoglobulin M were negative (Table 1). The peripheral blood smear did not demonstrate circulating blasts, atypical lymphocytes, or hairy cells. In conjunction with the absence of clinically significant lymphadenopathy on cross-sectional imaging, these findings reduced the likelihood of an overt leukemic or circulating lymphoproliferative process; definitive diagnosis, however, required examination of splenic tissue with immunophenotypic characterization.

**Table 1:** Baseline laboratory investigations The patient demonstrated significant leukocytosis with neutrophilic predominance and elevated lactate dehydrogenase, erythrocyte sedimentation rate, and C-reactive protein. The absolute lymphocyte count was preserved despite a reduced lymphocyte percentage. Peripheral blood smear demonstrated normocytic normochromic red blood cells and mature neutrophilic leukocytosis without circulating blasts, atypical lymphocytes, or hairy cells. Screening serological tests for HIV-1/2, hepatitis B surface antigen, anti-hepatitis C virus antibodies, Epstein-Barr virus IgM, and cytomegalovirus IgM were negative. HIV, human immunodeficiency virus; IgM, immunoglobulin M.

Parameter	Patient value	Reference range/expected result	Interpretation/clinical significance
Hemoglobin	12.2 g/dL	12.0-16.0 g/dL	Within the reference range; no significant anemia
Total leukocyte count	21,000/mm³	4,000-11,000/mm³	Elevated; significant leukocytosis
Neutrophils	78% (16,380/mm³)	40%-75% (1,500-7,500/mm³)	Elevated; neutrophilic leukocytosis
Lymphocytes	14% (2,940/mm³)	20%-40% (1,000-4,000/mm³)	Reduced percentage with preserved absolute count; relative lymphopenia
Monocytes	5% (1,050/mm³)	2%-10% (200-1,000/mm³)	Percentage within reference range; borderline absolute elevation
Eosinophils	2% (420/mm³)	1%-6% (40-400/mm³)	Percentage within reference range; borderline absolute elevation
Basophils	1% (210/mm³)	0%-2% (0-100/mm³)	Percentage within reference range; mild absolute elevation
Lactate dehydrogenase	480 U/L	140-280 U/L	Elevated (~1.7× upper limit of normal); nonspecific marker of cellular turnover/tissue injury and relevant to lymphoma assessment
Erythrocyte sedimentation rate	45 mm/hour	<20 mm/hour	Elevated; nonspecific marker of systemic inflammation
C-reactive protein	24 mg/L	<5 mg/L	Elevated; indicates systemic inflammatory activity
Peripheral blood smear	Normocytic normochromic red cells; mature neutrophilic leukocytosis	No abnormal circulating cells	No circulating blasts, atypical lymphocytes, or hairy cells identified
HIV-1/2	Negative	Negative/nonreactive	No serological evidence of HIV infection; relevant when evaluating lymphoma in the setting of immunodeficiency
Hepatitis B surface antigen	Negative	Negative/nonreactive	No serological evidence of current hepatitis B infection; relevant before immunochemotherapy
Anti-hepatitis C virus (HCV)	Negative	Negative/nonreactive	No serological evidence of HCV infection
Epstein-Barr virus (EBV) IgM	Negative	Negative/nonreactive	No serological evidence of recent EBV infection
Cytomegalovirus (CMV) IgM	Negative	Negative/nonreactive	No serological evidence of recent CMV infection

Abdominal ultrasonography demonstrated gross splenomegaly extending from the left upper abdomen into the pelvis (Figure 1).

**Figure 1:**
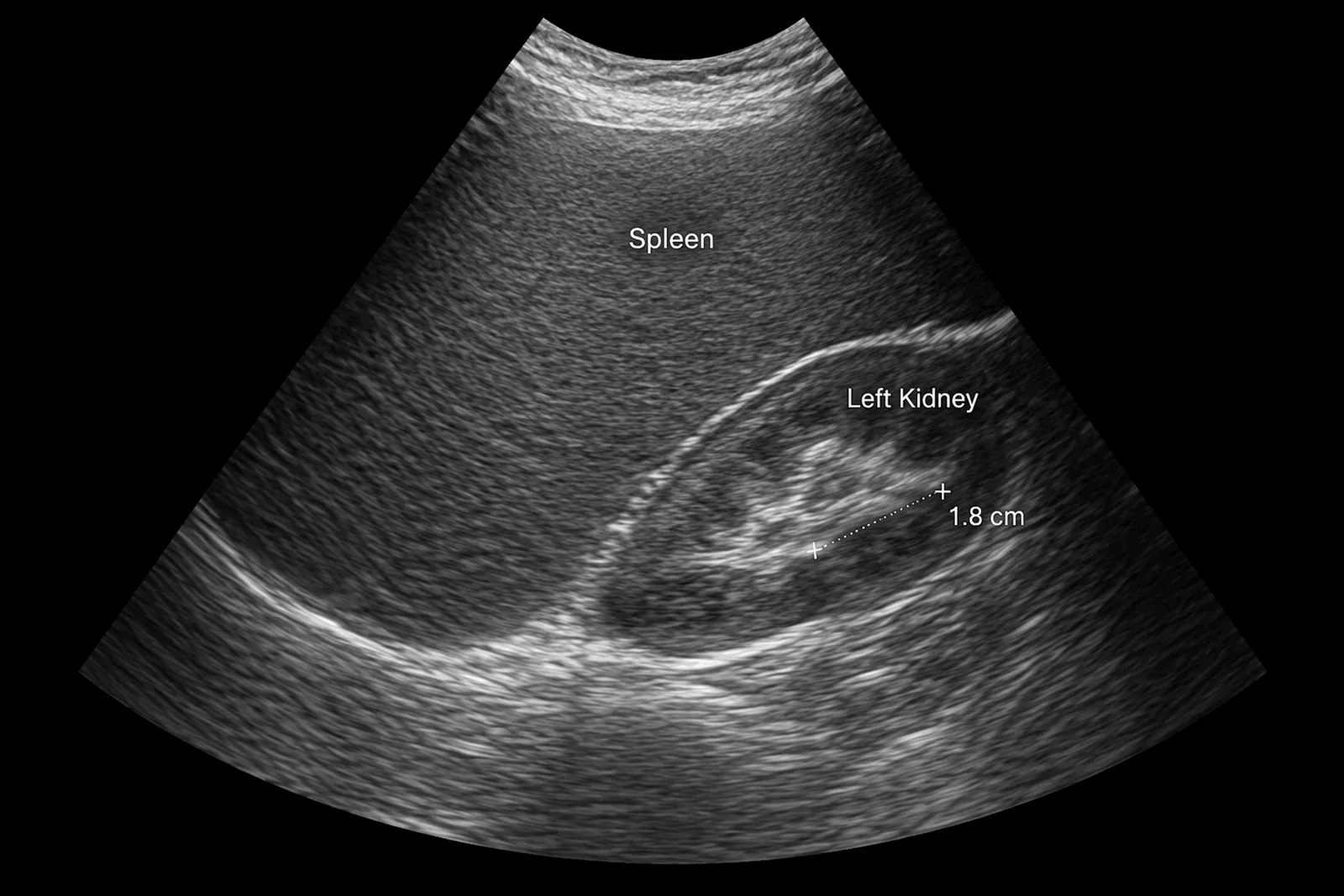
Ultrasonographic image demonstrating massive splenomegaly. Abdominal ultrasonography demonstrates marked enlargement of the spleen. The enlarged spleen occupies a substantial portion of the left upper abdomen and extends inferiorly. The left kidney is visualized separately, with a measured cortical thickness of approximately 1.8 cm. The image illustrates the marked splenic enlargement and its anatomical relationship with the left kidney.

Contrast-enhanced computed tomography (CECT) of the abdomen demonstrated a massively enlarged spleen measuring approximately 37 × 25 × 15 cm, extending from the left hypochondrium across the midline toward the right iliac fossa (Figure 2). There was no significant abdominal or pelvic lymphadenopathy, hepatomegaly, or other intra-abdominal visceral abnormality. CECT of the chest showed no significant mediastinal, hilar, or axillary lymphadenopathy, and the lung fields were clear. The preoperative differential diagnosis included splenic lymphoma, chronic myeloproliferative disorders, hairy cell leukemia, splenic marginal zone lymphoma, and other hematological or infiltrative causes of massive splenomegaly.

**Figure 2:**
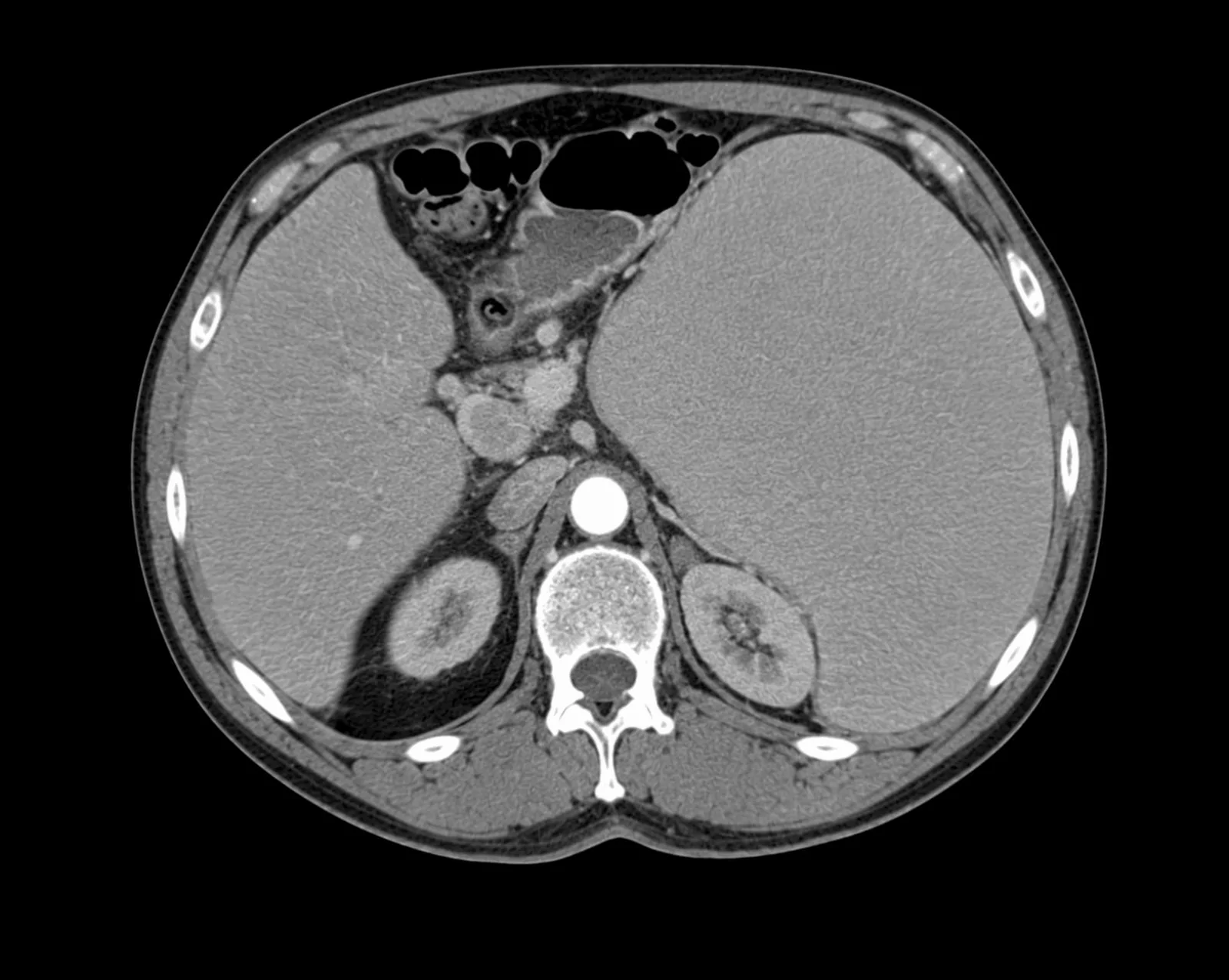
Contrast-enhanced computed tomography (CECT) of the abdomen demonstrated a massively enlarged spleen measuring approximately 37 × 25 × 15 cm, extending from the left hypochondrium across the midline to the right iliac fossa. There was no significant abdominal or pelvic lymphadenopathy, hepatomegaly, or other intra-abdominal visceral abnormality. Axial contrast-enhanced computed tomography of the upper abdomen demonstrating marked enlargement of the spleen, occupying a substantial proportion of the left upper abdominal cavity and extending inferiorly. The liver and both kidneys are visualized. No obvious enlarged abdominal lymph nodes are identified on this representative image. The image demonstrates the substantial mass effect produced by the enlarged spleen.

Given the extreme size of the spleen, progressive mass-effect symptoms, constitutional symptoms, and need for definitive tissue diagnosis, tissue acquisition was considered essential. Although image-guided splenic biopsy is an established diagnostic option in selected patients, the extreme splenic enlargement and distorted hilar anatomy raised concerns regarding the feasibility and potential bleeding risk of percutaneous biopsy in this patient. In view of the significant symptomatic burden and the requirement for definitive pathological diagnosis, elective open splenectomy was planned. The patient received preoperative vaccination against *Streptococcus pneumoniae*, *Neisseria meningitidis*, and *Haemophilus influenzae* type b 14 days before surgery using PCV13, MenACWY, and Hib vaccines, respectively, according to the institutional protocol.

An elective open splenectomy was performed through a midline incision. The operation lasted 155 minutes, with an estimated blood loss of 550 mL. Two units of packed red blood cells were transfused intraoperatively. The spleen was massively enlarged and occupied a substantial proportion of the abdominal cavity, with distortion of the hilar anatomy. Careful mobilization and vascular control were therefore performed (Figure 3). The splenic artery and vein were controlled with individual double ligation and suture ligation using nonabsorbable 2-0 silk. Complete splenectomy was achieved without injury to adjacent abdominal viscera or major vessels.

**Figure 3:**
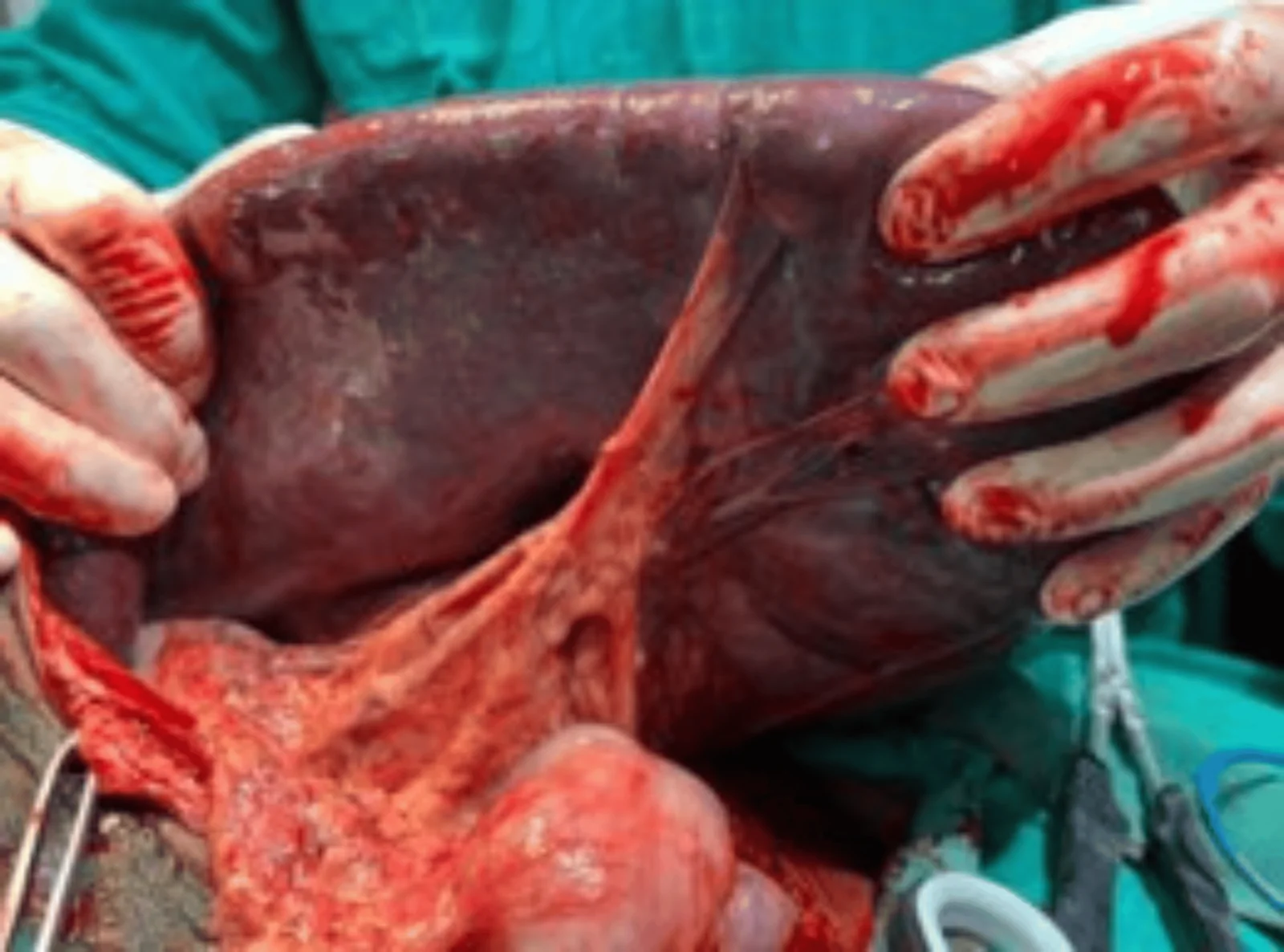
Intraoperative appearance of the massively enlarged spleen Intraoperative photograph demonstrating the markedly enlarged spleen during open splenectomy. The spleen is seen occupying a substantial portion of the operative field, with careful mobilization and dissection of the splenic attachments and hilar structures. The image highlights the technical challenges associated with surgical management of the massively enlarged spleen.

Gross examination of the specimen revealed a spleen measuring 40 × 27 × 12 cm and weighing 7.5 kg (Figure 4). The patient's recorded body weight was 43 kg, making the splenic mass approximately 17.5% of her total body weight.

**Figure 4:**
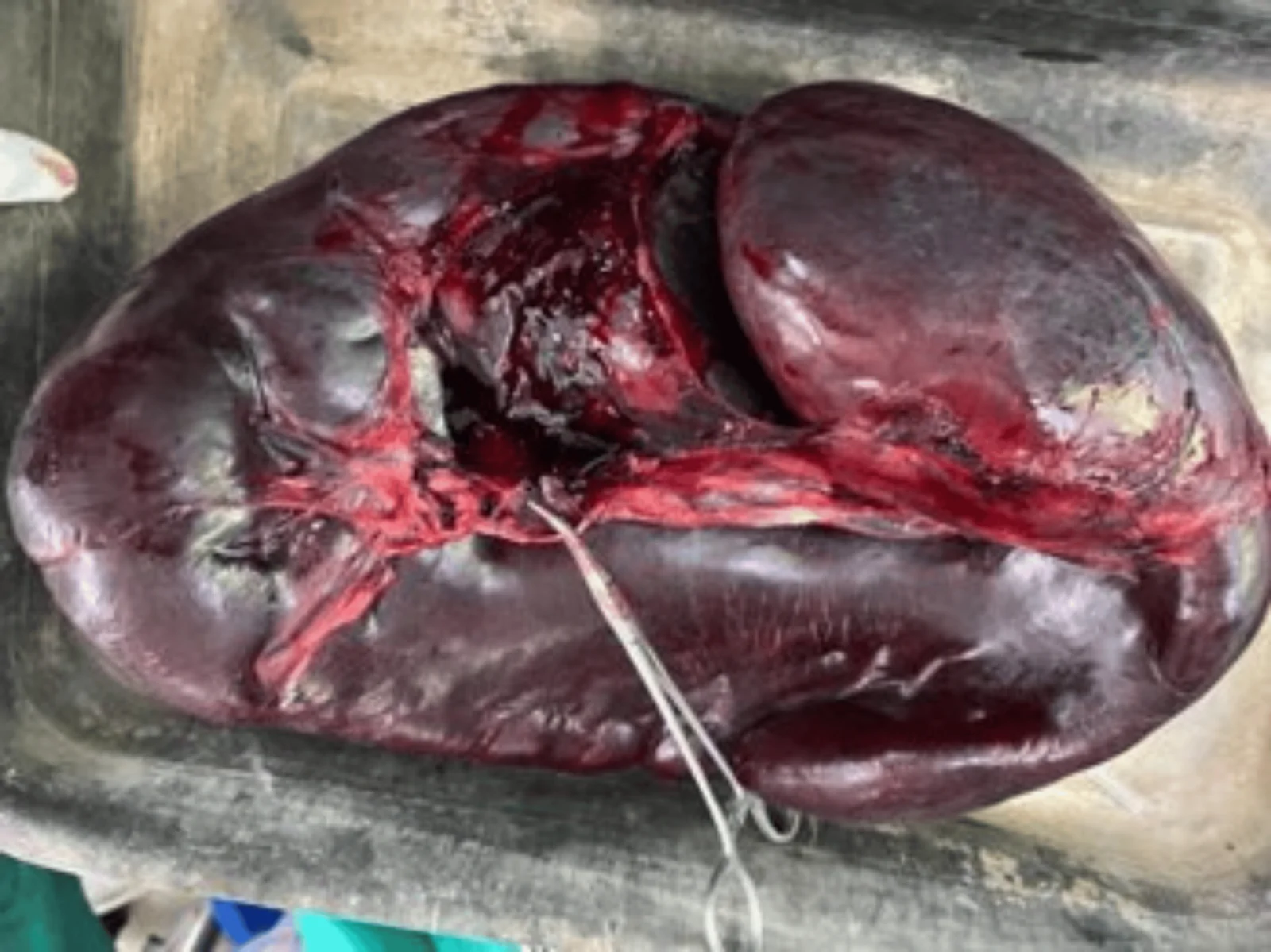
Gross specimen of the massively enlarged spleen Gross specimen of the spleen following open splenectomy, demonstrating marked splenomegaly. The spleen measured 40 × 27 × 12 cm and weighed 7.5 kg. The specimen represented approximately 17.5% of the patient's total body weight (43 kg), highlighting the exceptional degree of splenic enlargement.

Histopathological examination demonstrated diffuse effacement of the normal splenic architecture by sheets of large atypical lymphoid cells (Figure 5). The cells showed abundant eosinophilic cytoplasm, vesicular nuclei, irregular nuclear contours, conspicuous nucleoli, and frequent mitotic figures. No capsular or lymphovascular invasion was identified (Figure 6).

**Figure 5:**
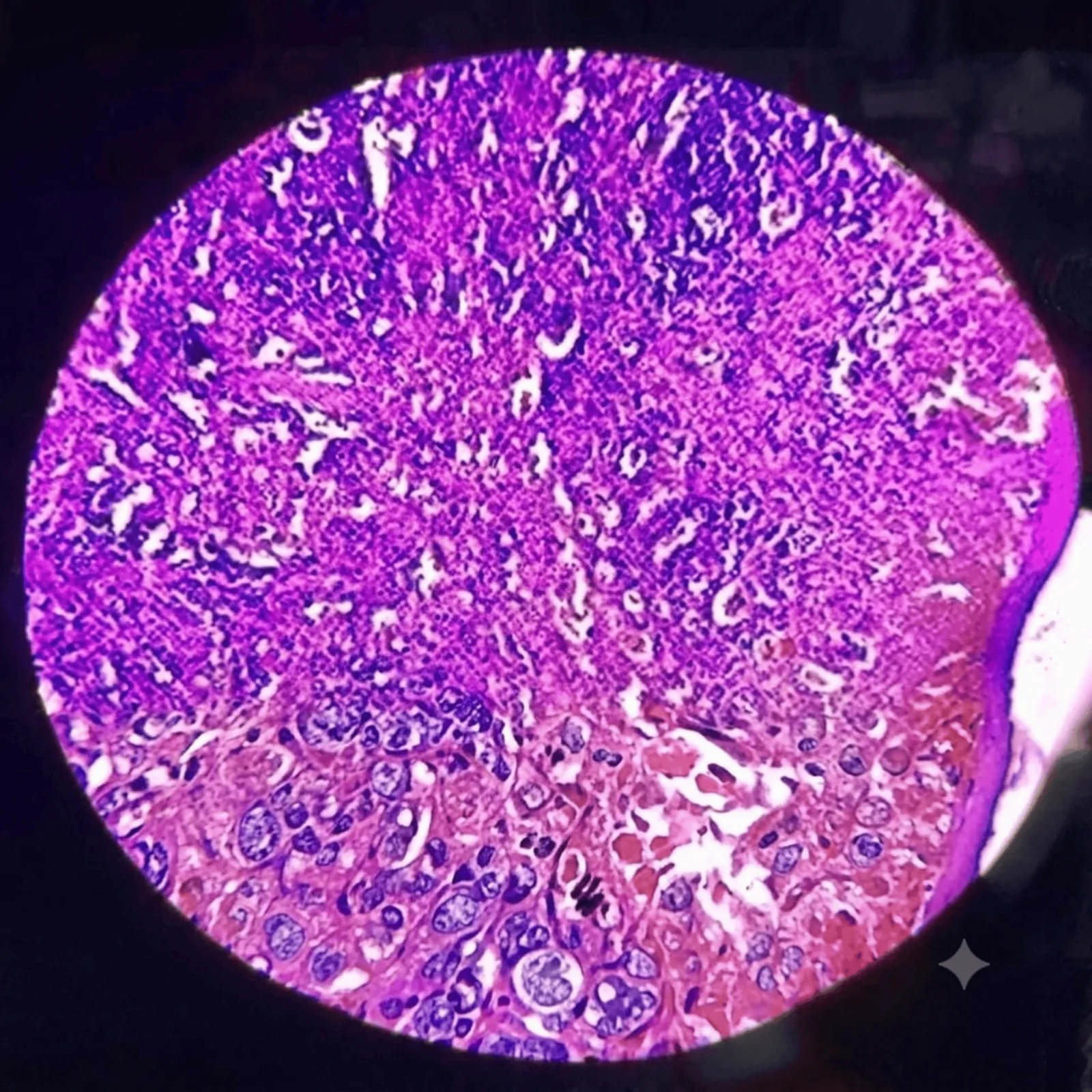
Histopathological examination of the splenic lesion Representative hematoxylin and eosin-stained section of the splenic lesion demonstrating diffuse effacement of the normal splenic architecture by a dense proliferation of large atypical lymphoid cells with vesicular nuclei, irregular nuclear contours, conspicuous nucleoli, and frequent mitotic figures. These findings are consistent with a high-grade lymphoid neoplasm and, together with the immunohistochemical findings, support a diagnosis of diffuse large B-cell lymphoma.

**Figure 6:**
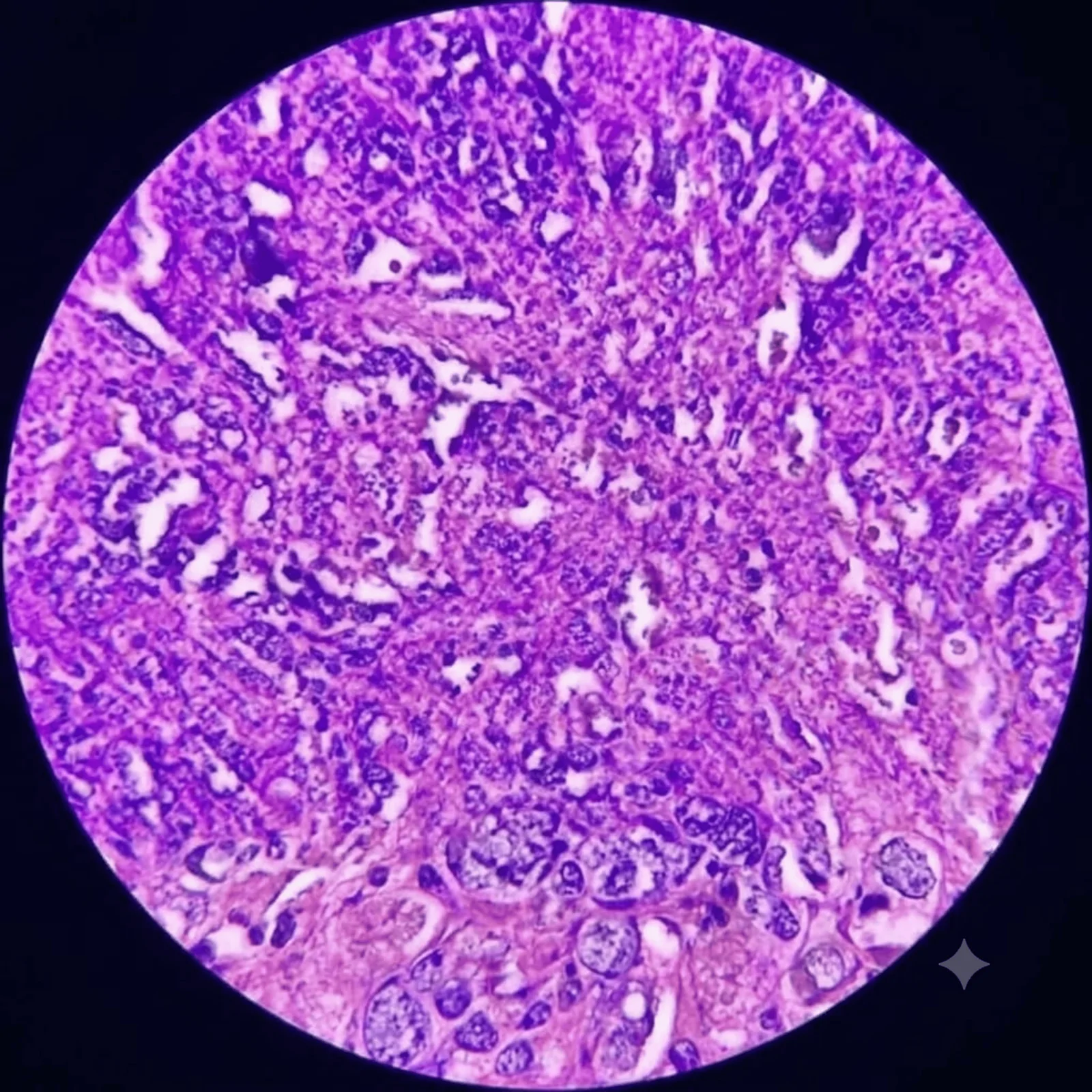
High-power histopathological examination of the spleen demonstrating diffuse large atypical lymphoid cells. High-power hematoxylin and eosin-stained section demonstrating diffuse proliferation of large atypical lymphoid cells with enlarged pleomorphic nuclei, vesicular chromatin, and prominent nucleoli, consistent with a high-grade lymphoid neoplasm.

Because immunohistochemical facilities were not available at the treating institution, representative paraffin-embedded tissue blocks were sent to an external laboratory for evaluation by an expert hematopathologist. Immunohistochemistry demonstrated positivity for the B-cell lineage markers CD20, CD79a, and PAX5, with negativity for CD3 and CD5. The Ki-67 proliferation index was approximately 80%. CD10 and BCL6 were positive, whereas MUM1 was negative, supporting a germinal center B-cell phenotype according to the Hans algorithm. BCL2 was expressed in fewer than 50% of tumor cells and MYC in fewer than 40%, below commonly used thresholds for a double-expressor phenotype (Figure 7). The combined morphological and immunophenotypic findings confirmed DLBCL of germinal center B-cell phenotype involving the spleen (Table 2). Additional molecular or flow-cytometric investigations for myeloproliferative or lymphoproliferative disorders were not performed because the diagnosis was subsequently established on splenic tissue with expert hematopathological immunophenotyping.

**Figure 7:**
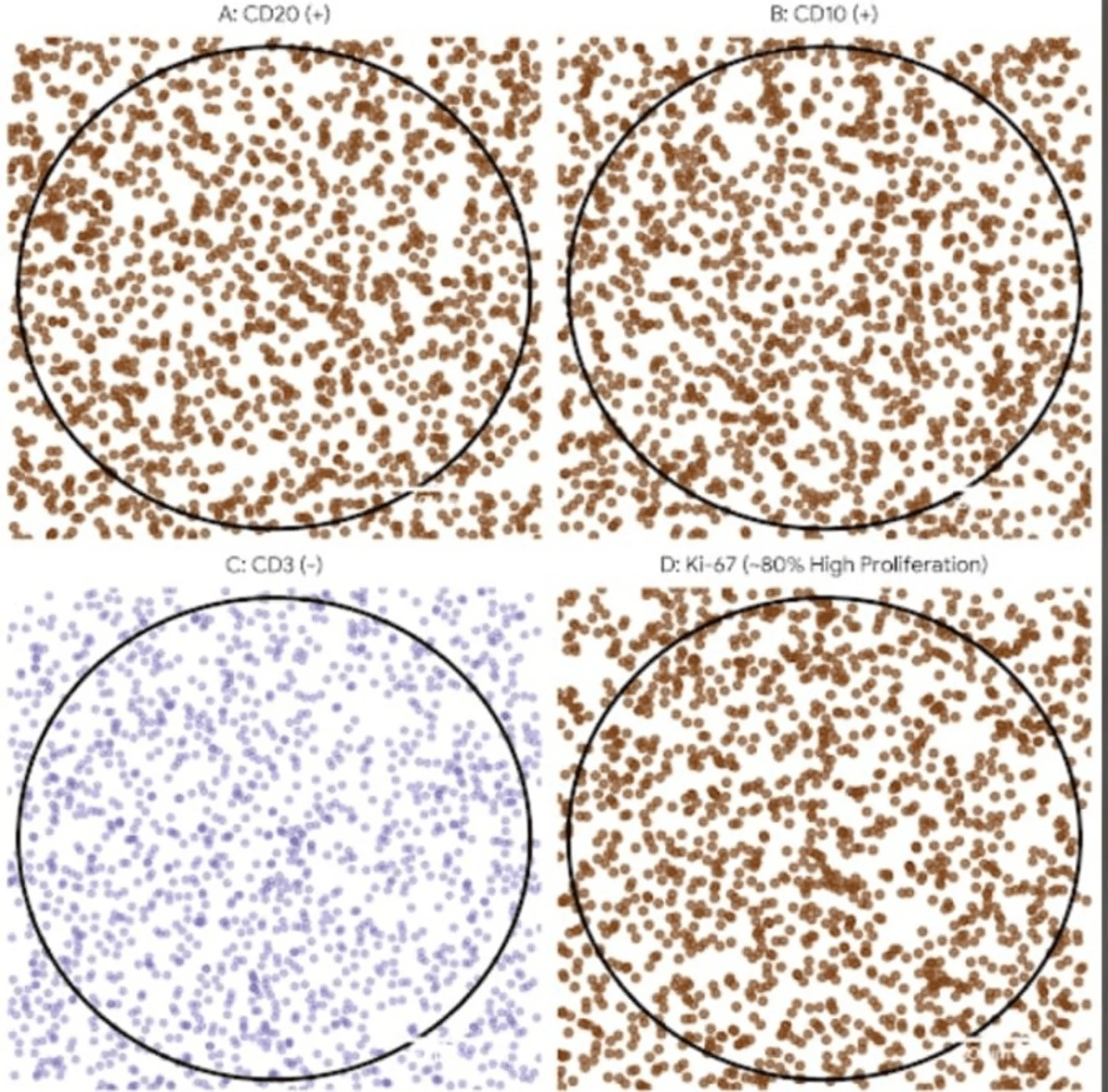
Representative immunohistochemical findings in splenic diffuse large B-cell lymphoma Representative immunohistochemical staining of the splenic tumor demonstrating the immunophenotypic profile of the neoplastic lymphoid cells. (A) CD20 shows diffuse positivity, supporting B-cell lineage. (B) CD10 shows diffuse positivity, supporting a germinal center B-cell phenotype. (C) CD3 is negative in the neoplastic cell population, excluding T-cell lineage. (D) Ki-67 demonstrates a high proliferative index of approximately 80%. CD, cluster of differentiation; Ki-67, marker of cellular proliferation.

**Table 2:** Immunohistochemical profile of the splenic lymphoma The immunohistochemical profile demonstrated expression of B-cell markers CD20, CD79a, and PAX5, with no expression of CD3 or CD5. CD10 and BCL6 positivity with MUM1 negativity supported a germinal center B-cell phenotype. The Ki-67 proliferation index was approximately 80%, indicating high proliferative activity. BCL2 and MYC expression remained below the predefined double-expressor thresholds. BCL2, B-cell lymphoma 2; BCL6, B-cell lymphoma 6; CD3, cluster of differentiation 3; CD5, cluster of differentiation 5; CD10, cluster of differentiation 10; CD20, cluster of differentiation 20; CD79a, cluster of differentiation 79a; IHC, immunohistochemistry; Ki-67, proliferation marker; MUM1, multiple myeloma 1; MYC, MYC proto-oncogene; PAX5, paired box 5.

Marker/assessment	Result	Interpretation
CD20	Positive	B-cell lineage
CD79a	Positive	B-cell lineage
PAX5	Positive	B-cell lineage
CD3	Negative	No T-cell lineage expression identified
CD5	Negative	No CD5 expression identified
Ki-67	Approximately 80%	High proliferative index
CD10	Positive	Germinal center phenotype
BCL6	Positive	Germinal center phenotype
MUM1	Negative	Supports germinal center phenotype
BCL2	<50% positive cells	Below the double-expressor threshold
MYC	<40% positive cells	Below the double-expressor threshold

The postoperative course was uncomplicated. The patient resumed oral intake as tolerated, and postoperative pain was adequately controlled. There was no postoperative hemorrhage, wound infection, or other major surgical complication. Mechanical thromboprophylaxis with graduated compression stockings was provided, along with pharmacological thromboprophylaxis using enoxaparin for the initial few postoperative days. Postoperatively, the platelet count increased from 285,000/mm³ on postoperative day 1 to 420,000/mm³ on day 3 and 650,000/mm³ on day 7, consistent with reactive thrombocytosis. The patient was discharged on postoperative day 10 with advice for appropriate follow-up.

Postoperative CECT of the chest, abdomen, and pelvis demonstrated no significant mediastinal, hilar, axillary, abdominal, or pelvic lymphadenopathy and no additional visceral disease, supporting a clinically localized splenic presentation. However, pretreatment 18F-FDG PET-CT and bone marrow assessment were not performed because of logistical and financial/resource limitations. Therefore, occult systemic or bone marrow involvement could not be definitively excluded. Because pretreatment PET-CT and bone marrow assessment were not performed, a definitive Ann Arbor/Lugano stage could not be assigned. Similarly, a complete International Prognostic Index (IPI) score could not be reliably calculated because formal disease stage and ECOG performance status were not documented; however, the patient's age was 47 years and serum LDH was elevated at 480 U/L (1.7 times the upper limit of normal). Because PET-CT was not performed, metabolic response and Deauville scoring could not be assessed.

Systemic treatment with R-CHOP immunochemotherapy was initiated 28 days after splenectomy. The patient completed six cycles administered at 21-day intervals. Treatment-related toxicity was limited to grade 1/2 alopecia and transient grade 2 neutropenia, without severe infection or treatment-related hospitalization. The patient underwent clinical, laboratory, and CT-based surveillance at 6, 12, and 18 months after completion of chemotherapy. At 18 months after completion of chemotherapy, she remained clinically well and asymptomatic, with no clinical or radiological evidence of recurrent disease on surveillance CT (Table 3).

**Table 3:** Clinical timeline of presentation, surgical management, systemic therapy, and follow-up The timeline summarizes the patient's clinical course from initial symptom onset through diagnosis, preoperative vaccination, open splenectomy, postoperative recovery, initiation and completion of chemotherapy with rituximab, cyclophosphamide, doxorubicin, vincristine, and prednisone (R-CHOP), and subsequent surveillance. CT, computed tomography; Hib, *Haemophilus influenzae* type b; MenACWY, meningococcal conjugate vaccine against serogroups A, C, W, and Y; PCV13, pneumococcal conjugate vaccine 13-valent; R-CHOP, rituximab, cyclophosphamide, doxorubicin, vincristine, and prednisone.

Time point	Clinical event
6 months before presentation	Onset of progressive left upper-quadrant pain and abdominal fullness
3 months before presentation	Weight loss, fatigue, fever, chills, and night sweats
At presentation	Examination revealed massive splenomegaly; laboratory and imaging evaluation were performed.
Preoperative period	PCV13, MenACWY, and Hib vaccination administered
Surgery	Open splenectomy performed; 7.5 kg spleen removed
Postoperative day 1-10	Uncomplicated recovery; reactive thrombocytosis monitored
Postoperative day 10	Discharged with oncology follow-up
28 days after surgery	R-CHOP initiated
Approximately 4.5 months after surgery	Six cycles of R-CHOP completed
6 months after chemotherapy	Clinical, laboratory, and CT surveillance
12 months after chemotherapy	Clinical, laboratory, and CT surveillance
18 months after chemotherapy	No clinical or radiological evidence of recurrence

## Discussion

PSL is an uncommon extranodal lymphoma, with DLBCL representing an important aggressive histological subtype. DLBCL is biologically heterogeneous, and accurate classification requires integration of clinical, morphological, and immunophenotypic findings [1]. In the present case, the disease manifested predominantly as massive splenomegaly with constitutional and abdominal symptoms and without clinically significant peripheral or thoracoabdominal lymphadenopathy.

DLBCL presenting predominantly as splenic disease may manifest with nonspecific constitutional and abdominal symptoms and may occur without generalized lymphadenopathy [2,3]. We observed similar findings in our patient, whose spleen measured 40 × 27 × 12 cm and weighed 7.5 kg. Isolated splenomegaly may occasionally be the principal or only clinically apparent manifestation of DLBCL [4]. The differential diagnosis of massive splenomegaly is broad and includes hematological malignancies, infections, infiltrative disorders, and other systemic conditions [5,6]. In our patient, the combination of constitutional symptoms, elevated LDH, neutrophilic leukocytosis, and extreme splenic enlargement increased suspicion for an underlying lymphoproliferative disorder. The absence of circulating blasts, atypical lymphocytes, or hairy cells on peripheral smear and the absence of clinically significant lymphadenopathy on cross-sectional imaging helped narrow the differential, although these findings alone could not establish the diagnosis.

The role of splenectomy in DLBCL presenting with massive splenic enlargement must be individualized. Previous reports have described splenectomy in patients with massive splenomegaly and significant hematological or mechanical manifestations [7]. In the present case, the spleen was exceptionally large, symptomatic, and associated with distorted hilar anatomy. Open splenectomy was therefore undertaken to obtain definitive tissue while simultaneously relieving the mechanical effects of the enlarged spleen. The procedure was completed with controlled hilar vascular dissection and without major visceral or vascular injury.

However, splenectomy is not universally required for diagnosis. Image-guided splenic core biopsy may provide adequate tissue in selected patients and can avoid the morbidity associated with diagnostic splenectomy [8]. The choice between percutaneous biopsy and splenectomy should therefore be individualized according to splenic size, vascular anatomy, lesion characteristics, procedural risk, severity of symptoms, amount of tissue required for comprehensive pathological classification, and institutional expertise. In this patient, the extreme organomegaly, persistent compressive symptoms, distorted hilar anatomy, and requirement for adequate definitive tissue favored an open surgical approach. Thus, the present case should not be interpreted as evidence that splenectomy is routinely preferable to image-guided biopsy in suspected splenic lymphoma.

Distinguishing a clinically isolated splenic presentation from secondary splenic involvement is also important. A tertiary-care series has demonstrated that both primary and secondary splenic lymphomas occur, emphasizing the importance of systemic assessment before assigning a primary splenic designation [9]. Postoperative CECT of the chest, abdomen, and pelvis in our patient showed no significant nodal or additional visceral disease, supporting a clinically localized splenic presentation. However, pretreatment 18F-FDG PET-CT and bone marrow assessment were not performed because of logistical and financial/resource limitations. Consequently, occult extra-splenic or bone marrow involvement could not be definitively excluded. For this reason, the disease in this patient is best described as DLBCL presenting as clinically isolated splenic disease rather than as definitively proven PSL.

Histopathological examination demonstrated diffuse replacement of the splenic architecture by large atypical lymphoid cells. Morphology suggested a high-grade lymphoid neoplasm but was not considered sufficient to establish B-cell lineage or definitively classify the tumor as DLBCL in the absence of immunophenotyping. Because specialized immunohistochemical facilities were unavailable at our institution, representative tissue was evaluated at an external laboratory by an expert hematopathologist. The neoplastic cells expressed CD20, CD79a, and PAX5 and were negative for CD3 and CD5, confirming B-cell lineage. CD10 and BCL6 positivity with MUM1 negativity supported a germinal center B-cell phenotype according to the Hans algorithm. The Ki-67/MIB-1 proliferation index of approximately 80% indicated high proliferative activity, while BCL2 and MYC expression remained below commonly used thresholds for a double-expressor phenotype. Thus, the diagnosis was established through integrated morphological and immunophenotypic assessment rather than morphology alone [10].

Systemic treatment remains essential following diagnosis of DLBCL because splenectomy alone does not address potential microscopic systemic disease. Published reports of splenic DLBCL have described systemic immunochemotherapy following tissue diagnosis [11]. Current ESMO Clinical Practice Guidelines recommend PET-CT for baseline staging and formal risk assessment in DLBCL and support R-CHOP-based systemic immunochemotherapy as a standard first-line treatment approach in appropriately selected patients [12]. In the present case, six cycles of R-CHOP were administered at 21-day intervals beginning 28 days after surgery. 

The principal limitation of this case is the absence of pretreatment 18F-FDG PET-CT and bone marrow assessment. Consequently, occult extra-splenic or marrow involvement could not be definitively excluded at diagnosis, and formal metabolic response assessment using PET-based criteria was not possible. The absence of PET-CT also precludes assignment of a Deauville score. Nevertheless, the absence of clinically significant lymphadenopathy or additional visceral disease on available cross-sectional imaging, together with definitive splenic histopathology and expert hematopathological immunophenotyping, supports the description of this case as a clinically localized splenic presentation of DLBCL.

This case highlights the diagnostic and therapeutic complexity of DLBCL presenting with exceptionally massive splenomegaly. In selected patients with extreme symptomatic splenic enlargement, open splenectomy may provide definitive tissue while simultaneously relieving mass-effect symptoms. However, surgery should be considered an individualized diagnostic and therapeutic intervention rather than definitive treatment for DLBCL. Image-guided core biopsy remains an important less-invasive alternative when technically feasible and clinically appropriate. Following histological confirmation, systemic lymphoma-directed therapy remains essential. In this patient, open splenectomy followed by six cycles of R-CHOP was associated with sustained clinical stability and no clinical or radiological evidence of recurrent disease during 18 months of follow-up. The absence of comprehensive pretreatment staging remains an important limitation and should be considered when interpreting the clinical outcome.

## Conclusions

DLBCL presenting as clinically isolated massive splenic disease should be considered in the differential diagnosis of patients with massive splenomegaly and constitutional symptoms, particularly when clinically significant peripheral or thoracoabdominal lymphadenopathy is absent. Definitive diagnosis requires adequate tissue examination and immunophenotypic characterization. Image-guided splenic biopsy may provide a less invasive diagnostic option in appropriately selected patients; however, in patients with extreme splenic enlargement, substantial mass effect symptoms, and unfavorable biopsy conditions, open splenectomy may provide both definitive tissue diagnosis and symptomatic relief. Systemic lymphoma-directed therapy remains essential after diagnosis. In the present case, the absence of pretreatment PET-CT and bone marrow assessment limited the ability to exclude occult systemic or marrow involvement. It precluded assessment of treatment response using PET-based metabolic criteria. At 18 months of follow-up after completion of chemotherapy, the patient remained clinically well with no clinical or radiological evidence of recurrent disease on CT-based surveillance.
